# Genes Contributing to Genetic Variation of Muscling in Sheep

**DOI:** 10.3389/fgene.2012.00164

**Published:** 2012-08-29

**Authors:** Ross L. Tellam, Noelle E. Cockett, Tony Vuocolo, Christopher A. Bidwell

**Affiliations:** ^1^Division of Animal, Food and Health Sciences, Commonwealth Scientific and Industrial Research OrganisationSt Lucia, QLD, Australia; ^2^Departments of Animal, Dairy and Veterinary Sciences, Utah State UniversityLogan, UT, USA; ^3^Departments of Animal Sciences, Purdue UniversityWest Lafayette, IN, USA

**Keywords:** sheep, skeletal muscle, gene, myostatin, Callipyge, imprinting

## Abstract

Selective breeding programs aiming to increase the productivity and profitability of the sheep meat industry use elite, progeny tested sires. The broad genetic traits of primary interest in the progeny of these sires include skeletal muscle yield, fat content, eating quality, and reproductive efficiency. Natural mutations in sheep that enhance muscling have been identified, while a number of genome scans have identified and confirmed quantitative trait loci (QTL) for skeletal muscle traits. The detailed phenotypic characteristics of sheep carrying these mutations or QTL affecting skeletal muscle show a number of common biological themes, particularly changes in developmental growth trajectories, alterations of whole animal morphology, and a shift toward fast twitch glycolytic fibers. The genetic, developmental, and biochemical mechanisms underpinning the actions of some of these genetic variants are described. This review critically assesses this research area, identifies gaps in knowledge, and highlights mechanistic linkages between genetic polymorphisms and skeletal muscle phenotypic changes. This knowledge may aid the discovery of new causal genetic variants and in some cases lead to the development of biochemical and immunological strategies aimed at enhancing skeletal muscle.

## Introduction

Significant improvement in sheep meat production has recently been made by using selective breeding and improved animal husbandry (Fogarty, 2009; Gardner et al., 2010). The breeding programs typically select sires based on production performance of their progeny, relatives and ancestors, or combinations thereof. Genetic selection and breeding using sires with high estimated breeding values (EBVs) for muscling traits is prevalent in the sheep meat industry.

DNA marker-assisted breeding strategies in sheep are now positioned to markedly accelerate the rates of genetic gain for desirable production traits, especially those that are difficult to measure, costly, and only expressed late in life. DNA markers in linkage disequilibrium (LD) with causal genetic variants are typically used in this strategy. Most livestock production traits are complex, involving contributions from a large number of additive genetic variants each of small effect size, interactions between genes, and strong gene by environment influences. To account for the additive contributions from many genes, and perhaps also epistatic effects, there has been recent focus on the implementation of genomic selection (or whole genome selection), which is “a form of marker-assisted selection in which genetic markers covering the whole genome are used so that all quantitative trait loci (QTL) are in LD with at least one marker” (Goddard and Hayes, 2007). A DNA marker-assisted selection strategy, and whole genome selection, will likely be more accurate and more robust if it incorporates contributions from causal genetic variations rather than relying entirely on markers in LD with these variants. Moreover, the discovery of causal variations can often suggest new opportunities for biochemical and immunological modulation of muscling traits. The latter strategy has potential for rapid implementation in all livestock and its impact is likely to be highly predictive and complement the use of genetic markers. These attributes are offset by its potential cost and the necessity for reintroduction of the application in each generation.

The rate of discovery of causal genetic variants (quantitative trait nucleotides or QTN) has been slow in most mammals. This is due to (i) the inability to efficiently dissect the broad genomic regions identified as containing QTN, (ii) the typically small effect sizes of most QTN, (iii) the difficulty providing conclusive proof, which often involves mechanistic investigation, and (iv) the absence, or only recent availability, of genome sequences for many domestic animal species (Ron and Weller, 2007; Braunschweig, 2010). The bovine genome sequence has been available since 2009 and an ovine genome sequence will likely be available in 2012 (Elsik et al., 2009; International Sheep Genomics Consortium et al., 2010). Genome sequences coupled with high density SNP chips provide opportunity to markedly accelerate discovery of causal genetic polymorphisms contributing to complex traits, like muscling. Despite the above limitations, a few causal genetic variants of relatively large effect sizes impacting on muscling traits have been discovered. Moreover, novel genetic, developmental, and biochemical mechanisms associated with these genetic variants have also been revealed.

The purpose of this review is to summarize this information, identify gaps in knowledge, and speculate on biochemical and immunological strategies for enhancement of muscling. Causal genetic polymorphisms are defined as those directly implicated in contributions to trait variation. In some instances independently confirmed genetic markers associated with skeletal muscle traits are also described, although the underlying causal polymorphisms are unknown. The impacts on eating quality traits associated with enhanced muscling in sheep have been recently summarized and are not discussed in any detail herein (Warner et al., 2010; Hopkins et al., 2011; Lambe et al., 2011).

## Skeletal Muscle form and Function

To understand the impacts of genetic variants affecting skeletal muscle traits in livestock, it is important to consider the normal functions and development of skeletal muscle. Skeletal muscle is superbly adapted to its functional roles. Muscles undergoing slow but continuous contractions in the post-natal environment, such as various postural muscles, are characterized by predominance of slow twitch fibers (type 1 fibers), which do not tire easily and are reliant on oxidative metabolism. They have abundant mitochondria and appear reddish in color. In contrast, muscles requiring rapid contraction generating substantial force, such as some locomotory muscles, have a greater proportion of fast twitch fibers (type IIb and IIx fibers). These muscles largely depend on glycolytic metabolism for energy generation and are whiter and have fewer mitochondria (MacIntosch et al., 2006). The number of fibers and fiber type composition are set during early to mid fetal development. During late fetal development and the rapid post-natal growth period there is extensive muscle fiber hypertrophy driven by fusion of mononuclear muscle satellite cells with multinucleated myofibers (MacIntosch et al., 2006). Satellite cells are located between the sarcolemma and basal membrane of muscle fibers, and while normally quiescent in adult muscle, upon muscle injury, they proliferate and fuse with myofibers thereby aiding repair. Muscle fibers are composed of a number of highly organized proteins, principally polymeric actin, myosin, and their ancillary proteins (Tellam, 1985; MacIntosch et al., 2006).

The transition from the ruminant fetal environment to that of the newborn is associated with major physiological changes. Skeletal muscle must rapidly adapt to meet the new demands of locomotion and postural support against gravity, whilst using different sources of energy than those available in the uterine environment. These adaptations are of great importance to newborn ruminants as they stand, walk, and run within a few hours of birth. Ovine skeletal muscle undergoes a major transition late in its development, which ensures newborn skeletal muscle is largely characterized by adult skeletal muscle fiber types (Byrne et al., 2010b). Thus, skeletal muscle is subject to a robust developmental program upon which is built responsiveness to environment cues. Mutations affecting muscling (i.e., muscle thickness relative to skeletal dimensions) typically alter the trajectory of this developmental program and change muscle fiber number, composition, and hypertrophy. Multiple ovine mutations have been discovered that enhance muscling and act through novel mechanisms impacting on this developmental program (Table 1). These mutations provide excellent models for studying muscle function and formation.

**Table 1:** **Characteristics of genes affecting muscling in sheep**.

Gene or QTL	Genomic location	Characteristics of QTN	Breed	Phenotypic effect (reference)
Myostatin	g + 6723G-A	SNP in the 3′ untranslated region of *MSTN* which generates an illegitimate miRNA binding site	Texel	Enhanced muscling and less fat (Clop et al., 2006; Johnson et al., 2009; Boman et al., 2010; Masri et al., 2011a,b)
Myostatin	g + 6723G-A	SNP in the 3′ untranslated region of *MSTN* which generates an illegitimate miRNA binding site	Charollais	Increased muscle depth (Hadjipavlou et al., 2008)
Myostatin	c.960delG	Frame-shift mutation	Norwegian White	Enhanced muscling and less fat (Boman et al., 2010)
Myostatin	Intron 1	–	Baluchi	Body weight (Ansary et al., 2011)
Callipyge	Telomeric region of OAR18	Intergenic SNP within an imprinted locus on the telomeric arm of OAR18 (phenotype only expressed post-natally by the paternal heterozygote)	American Dorset	Increased size of caudal muscles, leanness, shift toward type IIx and IIb fibers, increased FCE, increased toughness (Koohmaraie et al., 1995; Cockett et al., 1996, 2005; Jackson et al., 1997a,b,c; Freking et al., 1998a, 1999; Kerth et al., 1999; Siltberg and Liberles, 2002; Charlier, 2004; Vuocolo et al., 2007; White et al., 2007)
Carwell	2–6 cM telomeric of CSSM18 on OAR18	Not imprinted	Australian Poll Dorset	Increased depth of longissimus dorsi; no effect on fatness; possible change in muscle shape; shift toward IIb and IIx fibers (Nicoll et al., 1998; Greenwood et al., 2006)
LoinMax (rib eye muscling)	2–6 cM telomeric of CSSM18 on OAR18	–	Poll Dorset	Enhanced muscling in the loin (Masri et al., 2010)
TM-QTL	2 cM telomeric of CSSM18 on OAR18	Evidence for paternal expression of the muscling phenotype	British Texel	Enhanced muscling in the loin (Walling et al., 2004; Macfarlane et al., 2009, 2012; Rius-Vilarrasa et al., 2009; Matika et al., 2010)
Xinjiang	Telomeric region of OAR18	–	Xinjiang	Enhanced muscling (Liu et al., 2006)
QTL OAR1	2 QTL located on OAR1	Likely to be separate genes as QTL separated by 50 cM	Suffock and Charollais	Enhanced muscle depth or live weight (Walling et al., 2004; McRae et al., 2005; Matika et al., 2010)

## The Heritability of Muscling Traits in Production Sheep

Sheep muscling traits typically have moderate heritabilities. Various Merino and Border Leicester crosses have been examined for a variety of carcass and muscling traits (Mortimer et al., 2010). The ranges of heritabilities for muscle weight, meat yield, and carcass muscle dimensions were 0.22–0.35, 0.24–0.35, and 0.25–0.34, respectively. For United Kingdom Charollais, heritabilities for muscle depth and muscle depth corrected for live weight were 0.25 and 0.31, respectively (McRae et al., 2005), while heritabilities for muscle depth in Texel, Suffock, and Charollais sheep ranged between 0.38 and 0.54 (Matika et al., 2010). These values are similar to an average figure of 0.4, calculated for the muscling trait longissimus muscle area, in a meta-analysis of 72 studies in cattle (Utrera and Van Vleck, 2004). A number of QTL affecting muscling traits in various sheep populations have been discovered. Some of the underlying QTN have been identified, especially genetic variants with relatively large effect sizes, e.g., the myostatin (MSTN) and Callipyge mutations. Other QTL, usually of low to moderate effect sizes, have been confirmed in independent sheep populations but only localized to broad chromosomal regions containing many genes.

## Myostatin

Myostatin is a potent negative regulator of muscle mass in mammals. It is a member of the TGF-β superfamily of cytokines, several of which have key roles in maintaining skeletal muscle homeostasis through regulation of growth, differentiation, and regeneration of muscle (McPherron and Lee, 1997). Natural mutations in bovine, human, ovine, caprine, and canine *MSTN* that either inactivate the encoded protein or suppress its quantity cause enhanced muscling (Grobet et al., 1997; McPherron et al., 1997; Clop et al., 2006; Mosher et al., 2007; Stinckens et al., 2011; Zhang et al., 2012). Genetic manipulations that inactivate MSTN in transgenic mice and fish cause a similar phenotype (McPherron and Lee, 1993; McPherron et al., 1997; Sawatari et al., 2010). MSTN gene polymorphisms are also associated with racing speed for specific lines of horses and dogs (Mosher et al., 2007; Hill et al., 2010). For the cow, there are multiple mutations in *MSTN* that enhance muscling (McPherron and Lee, 1997). Most major skeletal muscles are affected by these mutations, which generally increase myofibril number (hyperplasia) and to a lesser extent myofiber cross-sectional area (hypertrophy), and increase the proportion of fast twitch glycolytic fibers. There are also decreased levels of connective and intramuscular adipose tissues (Bellinge et al., 2005). The impacts of the mutations are typically larger in homozygous animals compared with heterozygous animals. In sheep, several *MSTN* polymorphisms associated with muscling phenotypes have been reported (Table 1). The variety of different types of mutations that inactivate MSTN, particularly in cattle, are noteworthy and correspondingly there is a range of muscling phenotypes (Joulia-Ekaza and Cabello, 2006). Thus, there is a wealth of biological information accompanying MSTN mutations that is common to many mammalian species.

### Myostatin protein processing and signaling

**Figure 1:**
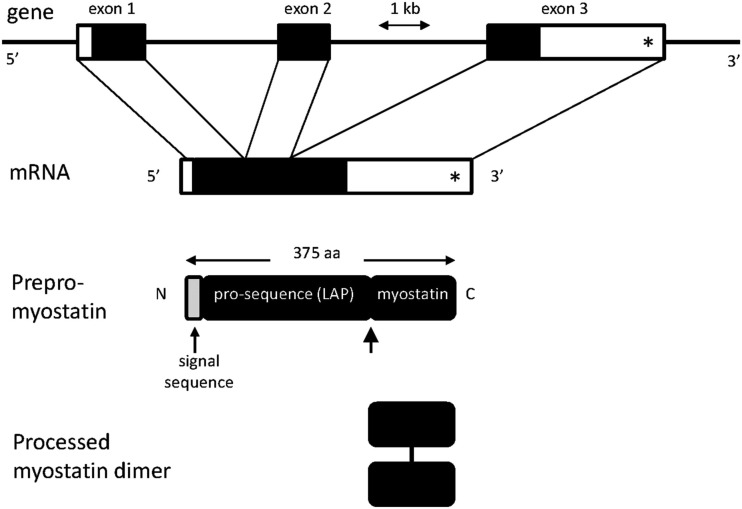
**Diagrammatic representations of myostatin (*MSTN*) gene and protein structures**. The gene consists of three exons. The position of the c. *1232 G > A mutation (previously referred to as g + 6723 G-A) is shown by an asterisk. Unshaded regions in exons and mRNA represent the 5′ and 3′ untranslated regions. The lightly shaded region in the prepro-myostatin protein sequence corresponds to the signal sequence. Also shown is the site for processing of the promyostatin protein (thick arrow). The mature myostatin polypeptide forms a dimer held together by a disulfide bond.

Myostatin is synthesized as a secreted 52 kDa precursor protein, which is proteolytically processed into the mature 12 kDa MSTN polypeptide and the 40 kDa N-terminal inhibitory propeptide (Latency Associated Peptide, LAP; Figure 1). Both the mature MSTN polypeptide (a dimer) and the latent inactive complex consisting of the MSTN polypeptide bound to LAP, circulate in blood (McPherron et al., 1997; Thies et al., 2001). Over-expression of LAP in mouse results in muscle mass enhancement. This is due to the ability of LAP to bind MSTN and inhibit its signaling activity (Li et al., 2010). Several circulating MSTN binding proteins [follistatin, decorin, small glutamine rich tetratricopeptide repeat (SGT), titan cap, Grb2-associating protein (GASP), follistatin related gene protein (FLRG)] have been identified in mouse. Over-expression of follistatin or FLRG increases skeletal muscle mass (Carnac et al., 2007). Presumably, these proteins sequester the mature MSTN polypeptide and thereby suppress its signaling capacity. Proof of this mechanism is still required for many of these proteins.

Myostatin binds to activin type II receptors (ACVR2A and ACVR2B) and activates the SMAD signaling transduction pathway resulting in altered transcription of a number of target genes. MSTN also activates various cyclin-dependent kinase pathways. This combined intracellular signaling leads to inhibition of the G1 to S transition in the cell cycle of myoblasts, inhibition of myoblast differentiation, and inhibition of satellite cell activation and renewal (Langley et al., 2002; McCroskery et al., 2003). Thus, the primary role of MSTN is to negatively regulate myogenesis.

Down-regulation or inhibition of MSTN activity during fetal development is thought to relieve MSTN’s suppression of muscle satellite cell activation and renewal, thereby promoting myofiber formation (Joulia-Ekaza and Cabello, 2006). This results in hyperplasia and to a lesser extent hypertrophy, with the latter caused by increased fusion of satellite cells with myotubes. The myofibers typically also have greater representation of type IIb fibers (Bellinge et al., 2005; Hennebry et al., 2009). It is noteworthy that the expression of murine *MSTN* is normally sixfold greater in predominantly fast versus slow twitch skeletal muscles suggesting that fast twitch myofibers may be more susceptible to inactivating MSTN mutations (Allen and Loh, 2011). Moreover, murine MSTN is a positive regulator of MEF2C, a key promyogenic transcription factor that normally promotes formation of slow oxidative fibers. MSTN is also a negative regulator of MYOD, another key promyogenic transcription factor with preferential expression in fast twitch glycolytic fibers. Thus, the absence of MSTN results in decreased MEF2C mediated slow twitch fiber formation and increased MYOD mediated fast twitch fiber formation (Hennebry et al., 2009).

### Recent selection for myostatin mutations

The MSTN gene, which encompasses three exons (Figure 1), and the signaling pathways through which it operates are conserved from zebrafish to humans, indicating that its functions are of ancient evolutionary origin (McPherron et al., 1997). Although mammals harboring MSTN mutations are usually viable, dystocia has been reported in some breeds of cattle (Keele and Fahrenkrug, 2001). The similar muscling phenotypes generated in widely different species carrying MSTN inactivating mutations also attest to the conservation of this muscle regulatory system. MSTN’s primary biological role may be to limit muscle size in a manner that is compatible with the size of the skeletal framework of each species.

The prevalence of mutations in the MSTN genes of cattle, sheep, goats, and to a lesser extent in racing dogs and horses, may reflect recent intensive artificial selection for muscling traits in these populations. Phylogenetic analysis measuring the ratios of non-synonymous to synonymous substitutions (Ka/Ks) indicates that the MSTN gene is subject to recent positive selection in the bovinae and caprinae–there is also some evidence for additional positive selection that predates domestication (Siltberg and Liberles, 2002; Tellgren et al., 2004; Bellinge et al., 2005). Moreover, the time to the most recent ancestor has been estimated at less than 400 years for a number of specific MSTN mutations, thereby inferring recent positive selection (O’Rourke et al., 2012). Recent, strong selection for MSTN has also been observed in three separate populations of Texel sheep (Kijas et al., 2012).

### Myostatin mutations in sheep

**Figure 2:**
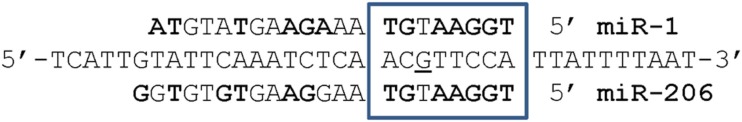
Alignments of the ovine miR-1 and miR-206 sequences with the region in the 3′ UTR of wild type ovine *MSTN* where the c. *1232 G > A mutation (underlined) occurs. Bolded nucleotides show complementarity of miR-1 and miR-206 with the *MSTN* 3′ UTR (center sequence). The eight nucleotide seed regions in the 5′ end of the miRNA are boxed. The G to A mutation produces an illegitimate recognition site for these miRNA and miRNA mediated translational repression of *MSTN* mRNA.

Texel sheep are characterized by enhanced muscling compared with other sheep breeds. In an elegant exemplification of modern genetic technologies, Clop et al. (2006) identified a QTL of major effect on muscling located in a 10 cM confidence interval on OAR2 using a Romanov × Texel F2 cross. The QTL accounted for 5–25% of the phenotypic variance. Fine mapping reduced the interval, which encompassed *MSTN*. Sequencing of the 10.5 kbp region of *MSTN* for Texel and control animals identified 20 SNP, most of which were monomorphic in Texel animals and consistent with a selective sweep across the locus. None of the SNP were located in the *MSTN* coding sequence. Analysis of offspring from an F2 ram that inherited an intact Texel chromosome and a recombined chromosome excluded 18 SNP. One of the two remaining SNP (c. *1232 G > A, but previously denoted g + 6723 G-A) was virtually Texel specific in the A allele and located in the 3′ UTR of *MSTN* mRNA (Figure 1). The variant created an illegitimate motif potentially recognized by three miRNA (*miR-1*, *miR-206*, and *miR-122a*; Figure 2). Two of the miRNA, *miR-1*, and *miR-206*, were known to be strongly expressed in skeletal muscle. Consistent with a miRNA mediated mechanism, the total level of circulating MSTN was about one third of that in wild type sheep, although the mRNA level in skeletal muscle was unaffected. Further experiments using reporter constructs transfected into COS1 cells unambiguously proved that the A allele created an illegitimate miRNA binding site in the 3′ UTR of *MSTN* mRNA allowing miRNA mediated translational repression. The effect of the mutation on muscling traits has been independently confirmed in a number of sheep breeds and crosses: Charollais, New Zealand Texel, Norwegian White, Texel × Poll Dorset cross, and Texel × Welsh Mountain cross (Hadjipavlou et al., 2008; Johnson et al., 2009; Boman et al., 2010; Masri et al., 2011a,b).

Additional mutations in the ovine MSTN gene affecting muscling have been described but in less detail. One frame-shift mutation (c.960delG) in Norwegian White Sheep resulted in increased muscling and less fat and these effects were stronger than in animals carrying the c. *1232 G > A mutation (Boman et al., 2009, 2010). There is also evidence for additional but as yet uncharacterized mutations possibly located in the promoter region, intron 1, intron 2, or 3′-UTR of *MSTN* in various sheep populations (Kijas et al., 2007; Hickford et al., 2010; Ansary et al., 2011).

Myostatin mutations have pleiotropic effects in cattle and this may also be expected in other mammalian species, including sheep. While increased muscularity and leanness are the most obvious phenotypes in ruminants, the mutation in cattle can also cause decreased sizes of several organs, fineness of limb bones, a higher incidence of underdeveloped genitalia, enlarged tongues, reduced fertility, low calf viability, increased stress susceptibility, and dystocia (Bellinge et al., 2005). These broad phenotypic effects suggest perturbation of normal system-wide development, which is most likely initiated in the embryo or early fetus, and accentuated during the period of rapid late fetal and post-natal growth. *MSTN* mRNA, while strongly expressed in skeletal muscle, is also expressed at lower levels in a number of adult tissues including adipose and mammary tissues. Therefore, inactivating *MSTN* mutations may directly affect other tissues, besides skeletal muscle (McPherron et al., 1997). In this regard, the change in body conformation and carcass “compactness” of sheep carrying the c. *1232 G > A *MSTN* mutation may be consistent with this concept (Boman et al., 2010; Masri et al., 2011b). Sheep carrying MSTN mutations are in widespread use in the sheep meat industry.

### Biochemical and immunological strategies to enhance muscling by suppression of myostatin activity

There are a number of strategies that might be employed to enhance muscling by exploiting the genetics and biological functions of MSTN. First, because MSTN is a negative regulator of myogenesis, biochemical, and immunological intervention strategies designed to directly suppress MSTN activity may enhance muscling. Indeed, immunization of mice and pigs against MSTN induced neutralizing humoral immune responses that enhanced muscling (Tang et al., 2007; Long et al., 2009; Zhang et al., 2011). A key to success of this approach may be the generation of sustained, neutralizing IgG responses. Other comparable approaches involve the use of antibodies blocking binding of MSTN to the activin type II receptors, dominant negative forms of the receptors, or dominant negative forms of the mature MSTN polypeptide (Siriett et al., 2007). In addition, exogenous LAP, follistatin, or FLGR could be used to sequester MSTN and enhance muscling, as demonstrated in mouse models (Carnac et al., 2007). In a variation on this strategy, histone deacetylase inhibitors could be used, as these have been shown to increase follistatin expression and enhance muscle repair in mice (Minetti et al., 2006). The limiting features of these approaches are likely to be consumer acceptability, duration of efficacy, cost, and frequency and timing of administration. These interventions may also need targeting to critical windows of fetal skeletal muscle development. Second, transgenic modifications using targeted mutagenesis of MSTN, RNAi directed at *MSTN* mRNA or over-expression of proteins that sequester MSTN can be envisaged (Tessanne et al., 2006). The remarkable quadrupling of skeletal muscle mass in a murine transgenic mouse engineered to have a *MSTN* knockout and over-expression of follistatin highlights the enormous plasticity of skeletal muscle and its potential for enhancement in livestock (Lee, 2007). Thus, direct interventions in MSTN signaling and its regulatory pathways are likely to have strong potential for enhancing muscling in livestock.

## Callipyge

The Callipyge mutation in sheep has provided remarkable new insights into genetics, regulation of gene expression and biology. The mutation causes skeletal muscle hypertrophy, but only in paternal heterozygous animals (N^mat^C^pat^; N, is the wild type allele and C is the allele carrying the mutation); a characteristic termed polar over-dominance (Cockett et al., 1996). The maternal heterozygote (C^mat^N^pat^) and homozygote (C^mat^C^pat^) animals show no muscling phenotypes. Specific skeletal muscles in the N^mat^C^pat^ genotype are increased in size by as much as 35%, although increased meat toughness has prevented commercial exploitation of these animals (Koohmaraie et al., 1995; Jackson et al., 1997a,b,c). In affected muscles, there is a marked shift toward type IIb and IIx fibers with a corresponding decrease in type I fibers, decreases in the proportional sizes of several non-muscle organs, increased leanness, and improved feed conversion efficiency (Jackson et al., 1997a,b,c; Charlier, 2004; Vuocolo et al., 2007; White et al., 2007). Live weight is unaffected and carcasses are more compact with a shorter length and greater width at the shoulder and rump (Freking et al., 1998a).

Unlike MSTN mutations, not all major muscles are affected by the Callipyge mutation. The muscling phenotype is first expressed 1–3 months post-birth and this occurs along a rostro-caudal gradient in the N^mat^C^pat^ animal, with greatest impact on skeletal muscles innervated through lumbar and sacral roots, e.g., *longissimus dorsi* and *semimembranosus* muscles (Koohmaraie et al., 1995; Freking et al., 1998a,b, 1999; Kerth et al., 2003; Cockett et al., 2005). As a consequence of the post-natal development of the phenotype, there is no increased risk of dystocia in N^mat^C^pat^ lambs. The muscling phenotype is maintained throughout adult life.

### Changes in expression of imprinted genes surrounding the Callipyge mutation

**Figure 3:**
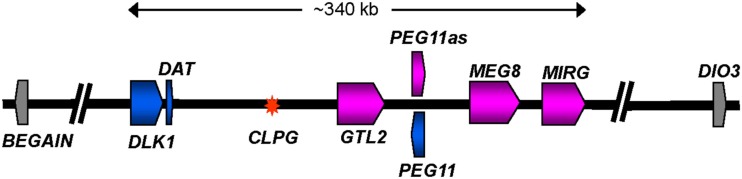
**Diagrammatic representation of the organization of imprinted genes located at the telomeric end of ovine chromosome 18**. The diagram shows a representation of the ∼1 Mbp region from *BEGAIN* to *DIO3*. The core imprinted genes affected by the Callipyge mutation are colored while imprinted genes unaffected by the mutation are shown in gray. Affected paternally and maternally expressed genes are shaded blue and pink, respectively. The direction of transcription for each gene is indicated by an arrow. Introns are not shown. The asterisk denotes the position of the Callipyge mutation (*CLPG*). The precise lengths of the maternally expressed genes, which all produce non-coding RNA, are unclear. The diagram is based on that deduced by (Georges et al., 2003) supplemented with annotation for a miRNA cluster (*MIRG*) deduced by comparative sequence analyses with orthologous murine and human sequence regions. A larger population of miRNA scattered throughout the core region has been omitted for clarity (Caiment et al., 2010).

The causative point mutation (A/G) responsible for the Callipyge muscling phenotype has been located in a 12 bp conserved motif positioned near the telomeric end of OAR 18 between the protein encoding gene *DLK1* (delta-like 1) and the non-protein encoding gene *GTL2* (gene trap locus 2; or *MEG3*; Figure 3; Freking et al., 2002; Smit et al., 2003). The ram in which the mutation first arose was a germ line mosaic for the mutation (Smit et al., 2003). The broader ∼1 Mbp region defines a cluster of conserved imprinted genes (Figure 3; Schmidt et al., 2000; Charlier et al., 2001a,b; Seitz et al., 2003; Takeda et al., 2006; Byrne et al., 2010a). The expression patterns of the core group of these imprinted genes are strikingly perturbed in affected skeletal muscles from N^mat^C^pat^ lambs, but are normal in unaffected muscles or the other genotypes (Bidwell et al., 2001, 2004; Charlier et al., 2001a; Murphy et al., 2005; Vuocolo et al., 2005, 2007; Fleming-Waddell et al., 2007, 2009; White et al., 2007; Caiment et al., 2010). Thus, altered gene expression at this imprinted locus is tightly linked with emergence of the phenotype. The affected genes span a core region of ∼350 kbp and include *DLK1*, *GTL2*, *PEG11* (paternally expressed gene 11; also known as retrotransposon like 1 (*RTL1*)), *PEG11AS* (antisense to PEG11; also known as *RTL1AS*), *MEG8* (maternally expressed gene 8) and *MIRG* (micro RNA-containing gene). While the expression of these genes is altered, their imprinted status remains unchanged. *DLK1* and *PEG11* are paternally expressed and both encode proteins, while the remainder are maternally expressed non-coding genes. The latter are all expressed from the same strand. *GTL2* expresses a large number of splice variants of a long non-coding RNA. *MIRG* encodes at least 50 miRNA, while *MEG8* encodes a cluster of CD snoRNA. As the muscling phenotype is only expressed in N^mat^C^pat^ animals, it was reasoned that the markedly enhanced expression of one (or both) of the paternally expressed protein encoding genes, *DLK1* or *PEG11*, was the primary driver of the muscling phenotype (Charlier, 2004). The mutation also enhances expression of the maternally expressed genes in N^mat^C^pat^ animals (Vuocolo et al., 2005). However, these effects are relatively small compared with their enhanced expression in the C^mat^N^pat^ and C^mat^C^pat^ genotypes, both of which do not express a muscling phenotype. Hence, these genes are unlikely to be directly promoting the phenotype.

It has been postulated that normal post-natal down-regulation of imprinted genes acts to coordinate the post-natal growth deceleration in many tissues (Lui et al., 2008). The Callipyge mutation in N^mat^C^pat^ animals prevents the normal post-natal down-regulation of *DLK1* and *PEG11* in skeletal muscle and recapitulates their fetal-like gene expression programs (Murphy et al., 2005; White et al., 2007). The dosage of these imprinted genes may be having major effects on the developmental program, and indeed for murine *Dlk1* there is evidence for an optimum dosage that fixes the upper limit of its growth promotion (da Rocha et al., 2009). There was no effect of the Callipyge mutation on the expression ratio of the two major *DLK1* splice variants in sheep skeletal muscle, even though there is a strong developmental switch in their usage (Vuocolo et al., 2005, 2007). The switch occurs during early to mid fetal development and long before the expression of the Callipyge phenotype a few weeks after birth. The splice variation produces a shorter plasma membrane-bound protein (predominant in the post-natal state) and a longer membrane-bound form (predominant in the early fetus). The latter is subject to proteolytic processing releasing a circulating form of DLK1 (Vuocolo et al., 2003).

### The primary effector gene driving muscle hypertrophy in Callipyge sheep

There is significant evidence indicating that DLK1 is the effector of the muscle hypertrophy phenotype in Callipyge sheep. First, the increased expression of *DLK1* as a function of genotype, development, muscle type, and muscle fiber type is strongly associated with the expression of the hypertrophy phenotype (Charlier et al., 2001a; Perkins et al., 2006; Fleming-Waddell et al., 2007; Vuocolo et al., 2007; White et al., 2007). In particular, this type 1 membrane-bound glycoprotein has increased expression in hypertrophied type IIb muscle fibers of the N^mat^C^pat^ genotype (Charlier et al., 2001a; Charlier, 2004; White et al., 2007). Second, transgenic mice over-expressing *Dlk1* using a myosin light chain 3F promoter were characterized by mild skeletal muscle hypertrophy (Davis et al., 2004). Third, DLK1 has been implicated in the control of proliferation and differentiation of many cell types (Wang and Sul, 2009) and is thought to be an atypical repressive ligand for the NOTCH signaling pathway. This is a key pathway negatively regulating cell commitment and differentiation (Vuocolo et al., 2007). Fourth, N^mat^C^pat^ myoblasts grown in cell culture and induced to differentiate do not become hypertrophic, but they also do not express *DLK1* (Lavulo et al., 2008). Fifth, consistent with the identification of DLK1 expressing mononucleated PAX7^+^ cells (muscle satellite cells) in fetal N^mat^C^pat^ skeletal muscle, over-expression of *Dlk1* in murine skeletal muscle satellite cells inhibited their proliferation and enhanced differentiation into multinucleated myotubes (White et al., 2007; Waddell et al., 2010). Sixth, genetic ablation of *Dlk1* in the murine myogenic lineage caused reduced body weight, reduced skeletal muscle mass, and impaired muscle regeneration, and in cultured satellite cells ablation promoted satellite cell self-renewal (Waddell et al., 2010). Over-expression of DLK1 on the surface of the multinucleated myotubes in some post-natal N^mat^C^pat^ skeletal muscles may be driving the characteristic hypertrophy of these muscle fibers. One model involves over-expression of DLK1 by nascent or regenerating myofibers which promotes the differentiation of neighboring satellite cells thereby leading to muscle hypertrophy (Waddell et al., 2010).

Despite the strong evidence for a direct role of DLK1 in initiating hypertrophy there is inconsistent information. First, a murine *Dlk1* knockout resulted in obesity but no reported effect on muscling (Moon et al., 2002). Second, constitutive expression of *Dlk1* in the murine myogenic cell line, C2C12, which does not express endogenous *Dlk1*, did not promote differentiation into multinucleated myotubes (Smas and Sul, 1993). Third, constitutive over-expression of *Dlk1* using its endogenous regulatory elements in transgenic mice resulted in growth enhancement but the mice failed to survive early life and showed no reported signs of muscle hypertrophy (da Rocha et al., 2009). These contradictory results may reflect the use of different experimental species and models and, in some cases, the lack of primary focus on skeletal muscle phenotypes. Species specific differences seem an unlikely explanation since the imprinted gene locus is highly conserved in placental mammals.

The paternally expressed gene *PEG11* is an alternative candidate for the effector. *PEG11* is a conserved Ty3-Gypsy retrotransposon like gene with a single long open reading frame encoding a 1,333 amino acid protein with retroviral gag-pol type structure. The gene is not associated with long terminal repeats and many of its encoded retroviral-like protein domains contain mutations that collectively destroy retrotransposon activity (Byrne et al., 2010a). The conservation of *PEG11* in placental mammals, and its absence from syntenic chromosomal regions of non-placental mammals, suggests that during evolution the newly retrotransposed gene was co-opted for novel functional roles within placental mammals (Lynch and Tristem, 2003). The full length PEG11 protein has been identified in nuclear fractions from Callipyge *semimembranosus* skeletal muscle (Byrne et al., 2010a). The biological role of PEG11 remains unclear although it has been implicated in angiogenesis during murine placentation (Sekita et al., 2008).

Like *DLK1*, the increased expression of *PEG11* as a function of genotype, development, and muscle type is also strongly correlated with the hypertrophy phenotype (Charlier et al., 2001a; Bidwell et al., 2004; Perkins et al., 2006; Vuocolo et al., 2007; White et al., 2007). *PEG11* is up-regulated about 45-fold in affected skeletal muscles from N^mat^C^pat^ animals at 3 months of age. In comparison, *DLK1* is up-regulated about 6- to 10-fold (Charlier et al., 2001a; Bidwell et al., 2004; Vuocolo et al., 2005, 2007; Byrne et al., 2010a). As discussed below, the maternally expressed gene *PEG11AS* contains a number of miRNA that cause RNA-induced silencing complex (RISC)-mediated cleavage of the paternally expressed *PEG11* transcript (Davis et al., 2005). Thus, if PEG11 was the effector driving the muscling phenotype, there is an obvious mechanism that could explain the lack of phenotype in homozygote animals. The potential role of PEG11 in inducing the muscling phenotype could be directly tested by its over-expression in transgenic mouse skeletal muscle or myogenic cells. This could be achieved by direct over-expression of PEG11 or by suppression of *PEG11AS*, and hence suppression of miRNA targeting *PEG11*. It is also possible that over-expression of both PEG11 and DLK1 are required to induce muscle hypertrophy. This hypothesis could also be tested in similar ways in transgenic mice.

It is difficult to envisage how the putative effectors, PEG11 or DLK1, could be used in direct biochemical and immunological interventions to enhance muscling. Both putative effectors positively enhance muscling and hence any intervention must induce increased levels of these proteins and they must be specifically targeted to skeletal muscle to avoid potential effects in other tissues. While exogenous soluble DLK1 may be introduced into an animal, it is unclear whether it would affect muscling as DLK1 may need cell surface presentation to responsive muscle cells. The intracellular location of PEG11 precludes a similar approach. Various genetic manipulations within this imprinted locus in mice have dramatic effects on gene expression at the locus and typically result in embryonic or fetal lethality (da Rocha et al., 2009). Consequently, it is likely that any exogenous effector will need to be targeted to skeletal muscle and expressed in a specific developmental window.

### A model for the inheritance of the Callipyge phenotype

There are unusual genetic mechanisms associated with the inheritance of the Callipyge phenotype, as the hypertrophy phenotype is only expressed by N^mat^C^pat^ animals (Cockett et al., 1994; Carpenter et al., 1996; Jackson et al., 1997a; Freking et al., 1998a,b; Bidwell et al., 2004). An elegant genetic model has been developed to explain this observation (Charlier et al., 2001a; Georges et al., 2003). The model proposes that the balance between the activities of a paternally expressed effector and a maternally expressed *trans*-acting repressor dictates the phenotypic outcome. The latter may exert its effects by translational suppression of the effector. In the N^mat^C^pat^ genotype the paternally expressed effector gene is influenced by the mutation acting in *cis*, thereby causing its over-expression relative to the maternally expressed repressor. The excess influence of the effector over repressor in this genotype induces muscle hypertrophy. Conversely, for C^mat^N^pat^ animals, where the animals are phenotypically normal, the mutation acts in *cis* to increase expression of the maternally expressed repressor, but it has no influence as the effector is not expressed from the wild type paternal allele. In the homozygote the paternally expressed effector and maternally expressed repressor are both up-regulated, but the influence of the effector does not dominate the repressor, and therefore no change in phenotype is generated.

The nature of the trans-acting maternally expressed repressor in this model is unclear although it is noted that only non-coding RNA are maternally expressed from the core of this imprinted locus (Charlier et al., 2001a). These non-coding genes are strongly up-regulated in the C^mat^N^pat^ and C^mat^C^pat^ genotypes, both of which do not express muscling phenotypes. In this regard, the *PEG11AS* transcript encodes six miRNA, which cause RISC-mediated cleavage of *PEG11* (Davis et al., 2005). Hence, these miRNA are ideally placed to act as maternally expressed *trans*-acting repressors of paternally expressed *PEG11*. In this model PEG11 is the putative effector of the phenotype.

An alternative, but analogous mechanism involves some of the approximately 110 likely maternally expressed miRNA that map to the core of the imprinted domain, and in particular a subset of approximately 50 miRNA encoded by the maternally expressed non-coding gene, *MIRG* (Glazov et al., 2008; Caiment et al., 2010). Some *MIRG* miRNA are predicted to target *DLK1* and many of these miRNA are up-regulated in *cis* with the Callipyge mutation (Hagan et al., 2009; Caiment et al., 2010). Experimental definitions of the targeting specificities for these maternally expressed miRNA are required to substantiate this hypothesis. It will also be important to define whether these miRNA act by RISC-mediated cleavage or translational repression of *DLK1*. A counterpoint to this mechanism is that many of the miRNA in this imprinted region show substantially increased expression in the C^mat^N^pat^ and C^mat^C^pat^ genotypes, which display no muscling phenotype. This result implies the effector is not expressed and there are no other mRNA targets for these miRNA in skeletal muscle cells from animals with these genotypes, despite the number of miRNA expressed from this region and their predicted broad mRNA target specificities. One possibility is that the primary role of these miRNA may involve maintenance of imprinting status at this locus rather than regulating mRNA. Intriguingly, the Callipyge mutation acting in *trans*, also causes a small increase in the expression of these maternally expressed miRNA, as it does for *GTL2*, suggesting that there is much more to learn about the complex regulatory events occurring within this imprinted gene locus (Caiment et al., 2010).

### Mechanisms causing muscle hypertrophy in Callipyge sheep

Microarray analyses of affected skeletal muscles have been undertaken to gain insight into the mechanism underlying generation of the hypertrophy phenotype in Callipyge lambs (Fleming-Waddell et al., 2007; Vuocolo et al., 2007). In one model generated from these studies, increased expression of DLK1 on myofibrils suppresses the myogenic inhibitory effects of the NOTCH signaling pathway in adjacent satellite cells, possibly in conjunction with suppression of HDAC9 (histone deacetylase 9), a known negative regulator of myogenesis (Vuocolo et al., 2007). This promotes satellite cell activation and their fusion with adjacent multinucleated myotubes thereby generating larger hypertrophied myotubes. The discovery that DLK1 is expressed on the cell surface of a subset of mononuclear satellite cells and on the surface of multinuclear myofibers in N^mat^C^pat^ skeletal muscle supports this possibility (White et al., 2007; Waddell et al., 2010). Another investigation suggested that activation of the AKT/mTOR signaling pathway was the primary driver of enhanced protein synthesis in muscles affected by the mutation (Fleming-Waddell et al., 2009). Both AKT/mTOR and HDAC9 are known to be important regulators of myogenesis and muscle hypertrophy in mouse models (summarized in Fleming-Waddell et al., 2007, 2009; Vuocolo et al., 2007).

### Interplay between the Callipyge mutation and epigenetic marks

How does the Callipyge mutation act over considerable genomic distances to cause changes in gene expression within the core of the imprinted domain? A clue to the mechanism whereby the Callipyge mutation, acting in *cis*, increases expression of a number of adjacent imprinted genes can be found in changes to DNA methylation surrounding the site of the mutation. In post-natal N^mat^C^pat^ samples from affected, but not unaffected, muscles, the wild type allele is strongly methylated but the Callipyge allele is hypomethylated (Murphy et al., 2006). The mutation is also associated with development of novel DNase-I hypersensitivity sites, indicating increased relaxation of the chromatin state (Takeda et al., 2006). Consistent with this permissive epigenetic state, the mutation, acting in *cis*, causes bidirectional long range *DLK1*-*GTL2* intergenic transcription of non-coding RNA in the lamb (Takeda et al., 2006). The conserved 12 bp motif, in which the mutation is located, is normally likely to be a long range regulatory element, possibly an insulator or enhancer, that exerts developmental and tissue specific controls on the expression of multiple genes in the core of the imprinted region. The mutation is presumed to perturb these roles by changing binding affinity for key transcriptional co-regulators thereby preventing the normal program of post-natal down-regulation of imprinted genes in this region. The mutation also exerts influence in specific muscles toward the rear of the animal possibly through the actions of a rostro-caudal positionally graded regulator, whose pattern of expression is set early in embryonic development.

The polar over-dominant inheritance pattern characteristic of the Callipyge muscling phenotype is more widespread in mammals than widely appreciated. In pigs, the same locus is associated with growth, fatness, and body composition in a polar over-dominant manner (Kim et al., 2004; Oczkowicz, 2009). In humans, a SNP, rs1802710 situated in exon 3 of *DLK1*, is associated with polar over-dominant expression of child and adolescent obesity (Wermter et al., 2008). Another SNP, rs941576, located within *GTL2* is associated with paternally inherited risk of type 1 diabetes (Wallace et al., 2010). Notably, *Dlk1*-null mice show growth retardation, accelerated obesity, and hyperlipidaemia (Moon et al., 2002). A genome scan for imprinted body weight and growth QTL in mice has identified additional unusual inheritance patterns at a variety of loci elsewhere in the genome (Wolf et al., 2008). These patterns include bipolar over-dominance (the two heterozygotes differ from each other but the two homozygotes have similar phenotypes), and polar under-dominance (one of the two heterozygotes exhibits a lower intensity of phenotype compared to the other three genotypes). Widespread allelic imbalance in gene expression and DNA methylation are likely to be the norm and not the exception (Kong et al., 2009; Meaburn et al., 2010). Some of this imbalance will be due to allelic contributions and trans-acting epistatic effects, while other contributions are likely to reflect epigenetic based parent-of-origin effects and generalized monoallelic gene silencing. Unless expressly tested, these non-Mendelian effects are likely to have been missed in current genome-wide trait association analyses (Matika et al., 2010). Re-analysis of existing datasets using a broader range of genetic models is warranted, as this may reveal new QTL with non-Mendelian behavior and simultaneously enhance sensitivity to detect standard QTL.

## Confirmed Quantitative Trait Loci for Muscling in Sheep

In addition to highlighting the contributions of MSTN polymorphic variants to muscling phenotypes, several genome scans in different populations of sheep have identified QTL for muscling on the telomeric end of OAR 18. These are different from the effects of the Callipyge mutation, but located at the same general position (Cockett et al., 2005).

### Carwell, LoinMAX™ and other muscling QTL located on the telomeric arm of OAR18

The Carwell (or rib eye muscling, REM) locus identified in Australian Poll Dorset rams is associated with increased muscle depth of *longissimus dorsi* (also named *longissimus lumborum*) with no effect on fatness or live weight. There is also a change in muscle shape and a shift from type IIa towards type IIb and IIx muscle fibers (Nicoll et al., 1998; Greenwood et al., 2006). The QTL is positioned 2–6 cM telomeric to the CSSM18 marker on OAR18 and overlaps with the site of the Callipyge mutation. The effect of each allele was additive and dominant, and there was no indication that the QTL was imprinted; however, this possibility was not explicitly tested. The effects of this QTL are relatively small and more variable compared with Callipyge. Recently, a QTL (LoinMAX™) for the same muscling trait was located within this genomic interval in a cross between Poll Dorset heterozygous ram carriers and British cross-bred ewes (Masri et al., 2010). In this instance, there was a small extent of enhanced muscling and no effects on carcass lean weight, fat content or body conformation scores.

In Texel and Texel–Suffolk families another confirmed muscling QTL (TM-QTL) was localized to 2 cM telomeric of the same marker (CSSM18) on OAR18 (Walling et al., 2004; Macfarlane et al., 2009, 2012; Rius-Vilarrasa et al., 2009; Matika et al., 2010). The muscling phenotype in these animals was similar to that in the Poll Dorset sheep and associated with altered muscle shape–the carcass was described as more “compact.” It was recently concluded there is evidence for imprinting of this QTL. It showed a polar over-dominance model of inheritance suggesting similarity with the Callipyge mutation, albeit the muscling effect size of the QTL was much smaller (Matika et al., 2010). In a fourth example, a polymorphism in this same region was associated with muscling traits in Xinjiang sheep (Liu et al., 2006). It is likely that discernment of finer details of the muscling phenotypes and inheritance patterns in these four sheep populations is limited by the relatively small muscling effect sizes of these QTL.

The causal genetic polymorphisms linked with these QTL are unknown although they are unlikely to be directly related to the Callipyge mutation for the following reasons. (i) Direct examination of DNA sequence at the site of the Callipyge mutation does not reveal any sequence variation in the Texel or Xinjiang animals (Liu et al., 2006; Matika et al., 2010). (ii) The absolute effects on specific muscles were much weaker for these QTL (∼2.5–7% increase in muscling compared with up to 35% in some muscles from Callipyge sheep), and effects on fat depth and muscle tenderness were also quantitatively different. The pronounced rostro-caudal muscling gradient in Callipyge animals was also not apparent in these other populations. (iii) Callipyge has a complex inheritance pattern that was not readily apparent for all of these QTL, with the exception of the Texel QTL (Matika et al., 2010). Carwell shows a simple autosomal dominant pattern of inheritance (Jopson et al., 2001), although there has been an unconfirmed suggestion of sire-dependent effects in the progeny of LoinMAX™ animals (Nicoll et al., 1998). The pathway to commercial exploitation of these QTL will critically depend on whether or not they are imprinted, as this will have a strong influence on breeding flock design. A homozygote terminal sire would likely be used. All progeny from this sire would inherit one active copy of the imprinted gene because of the paternal transmission mechanism. QTL for muscle depth and live weight have been also located on OAR1 in Suffock and Charollais populations, respectively (Walling et al., 2004; McRae et al., 2005; Matika et al., 2010). The QTL lie 50 cM apart and are therefore unlikely to be caused by polymorphisms in the same genes. The corresponding QTN have not been defined.

## Integration of Gene Expression with Genetic Architecture Associated with Enhanced Muscling

Muscle accretion is the result of contributions from a number of developmental and biochemical pathways. Multiple genetic variants in these pathways, acting in concert, are likely to underpin enhanced muscling traits in most production populations, particularly in the absence of genetic variants of relatively large effect sizes, e.g., the Callipyge and MSTN mutations. The impacts of these multiple genetic variants, each of small effect size, can be measured through their combined influence on these pathways. Using Poll Dorset sheep, the pathways associated with the genetics of enhanced muscling were identified by linking gene expression patterns in progeny skeletal muscle with sire based EBVs for the muscling trait, *longissimus dorsi* depth (Kogelman et al., 2011). In this study there was strong sire based genetic structure associated with the gene expression program in progeny skeletal muscle. Higher sire EBV was also probably associated with a shift toward fast twitch glycolytic fibers in progeny. It was concluded that sires with high muscling EBVs were characterized by progeny muscle accretion mediated by activation of pathways that include enhanced myogenesis, muscle fiber hypertrophy, and decreased protein catabolism. Low EBV status resulted from increased genetic contribution to protein degradation pathways. As may be expected, the key genetic effects dictating EBV status impacted on the balance between muscle fiber protein accretion and turnover. This type of analysis, particularly if coupled with SNP association data, has potential to accelerate the identification of additional genetic variants contributing to muscling by the discovery of pathways directly contributing to muscling traits. The analysis may also be particularly suited to dissection of complex traits. One strength of this approach is that the application of RNA-seq could allow identification of genes demonstrating allelic expression imbalance. This is a signature for a *cis*-acting SNP regulating gene expression (excluding imprinted genes). These SNP could be directly contributing to the trait.

## Conclusion

A number of genetic variants with confirmed effects on muscling in sheep have been discovered. There are several common features associated with a high muscling phenotype in sheep irrespective of the primary genetic driver. (i) Enhanced muscling is often associated with morphology change of the animal. This suggests a common change in developmental programming that scales body shape to accommodate enhanced skeletal muscle structure. (ii) Changed developmental trajectories for skeletal muscle beginning in early life are also common and these may be linked with morphology changes. (iii) Large increases in muscularity are typically associated with a shift toward fast twitch glycolytic fibers, leanness, and poorer eating quality attributes. The reasons for these changes are unclear, although both myoblasts and preadipocytes have a common progenitor cell, suggesting a developmental link. (iv) The mutations associated with MSTN and Callipyge are typically recent and for MSTN there has been strong and positive selection in production flocks. (v) The co-occurrence in Texel sheep of a recent mutation in MSTN contributing to muscling, as well as a confirmed QTL for muscling on the telomeric end of OAR18, indicates that artificial breeding programs have enriched for genetic variants with independent and additive effects. This suggests the involvement of fundamental and independent regulatory mechanisms that presumably normally function to limit muscle growth.

The immediate future of the sheep meat industry will, see the increasing exploitation of natural genetic variation contributing to muscling. The incorporation of causal genetic variations into genomic selection strategies will enhance their accuracy and robustness, while allowing targeted selection to achieve more rapid genetic improvement. In the medium term, the discovery of developmental and biochemical pathways contributing to enhanced muscling will open new opportunities for the use of novel and acceptable biochemical and immunological interventions that may play a significant and complementary role to genetic selection in the sheep meat industry.

## Conflict of Interest Statement

The authors declare that the research was conducted in the absence of any commercial or financial relationships that could be construed as a potential conflict of interest.
